# Supplementary material for: Extreme ultraviolet imaging of three-dimensional magnetic reconnection in a solar eruption
Source: Nat Commun. 2015 Jun 26;6:7598. doi: 10.1038/ncomms8598 (PMC4491808; doi:10.1038/ncomms8598)
Supplement: Supplementary Figure — 1 [file ncomms8598-s1.pdf]

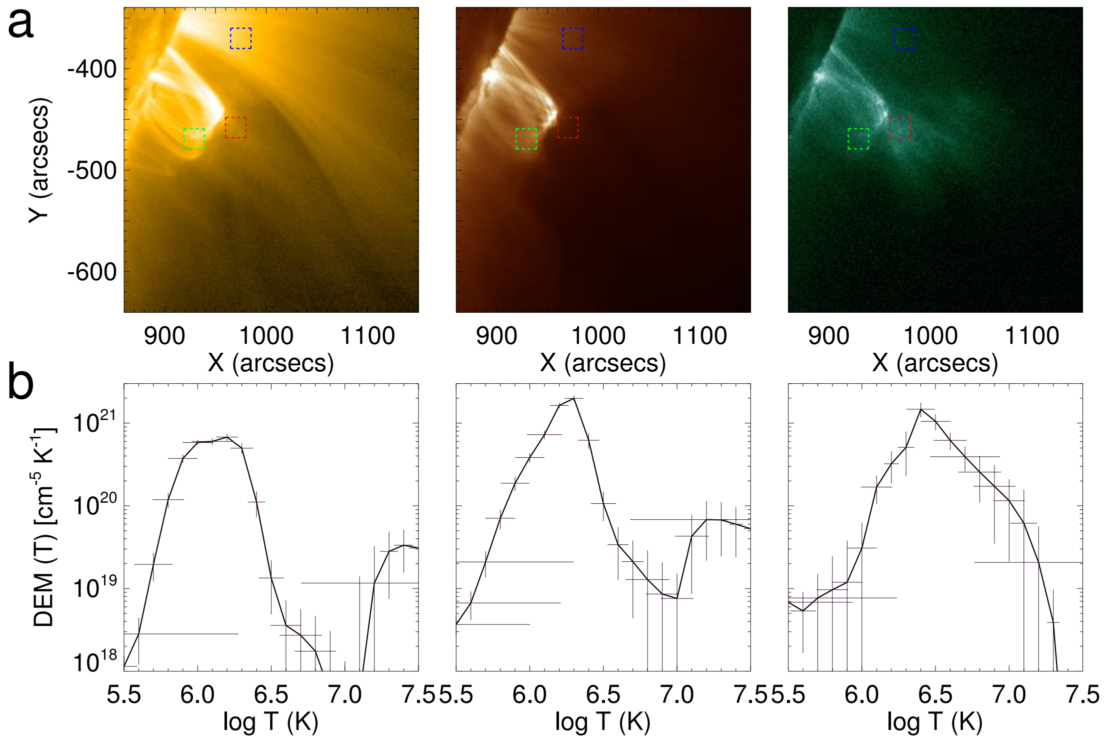

Supplementary Figure 1: DEM solutions of different coronal structures. (a) The AIA 171 Å, 193 Å, and 94 Å images at 08:14 UT. The three boxes display the three different regions that we use for DEM calculations. (b) DEM distributions of the quiet Sun region (blue), the flare loop (green), and the hot region (red). The horizontal bar represents the uncertainty of the temperature, i.e., temperature resolution. The vertical bar displays the error of the DEM, which denotes the deviation of Monte Carlo solutions from a real one.
